# Factors for not performing total body skin examinations in primary care in association with teledermoscopy

**DOI:** 10.1186/s12875-023-02034-4

**Published:** 2023-03-21

**Authors:** Jonas Ingvar, Kari Nielsen, Åsa Ingvar

**Affiliations:** 1 Dermatology and Venereology, Department of clinical sciences Lund, Lund University Skin Cancer research group (LuScaR), Lund University, Lund, Sweden; 2 Kry Laurentii Primary Care Center, Lund, Sweden; 3 Department of Dermatology, Skåne University Hospital, Lund, Sweden; 4 Department of Dermatology and Venereology, Helsingborg Hospital, Helsingborg, Sweden

**Keywords:** Teledermoscopy, Skin cancer, Total body skin examination, Physician gender

## Abstract

**Purpose:**

To investigate factors related to omitted total body skin examination (TBSE) in skin cancer diagnostics while managing patients using teledermoscopy (TDS) in Swedish primary care.

**Methods:**

4,987 TDS referrals from primary care centers were analyzed to identify factors associated with failing to perform TBSE. Data collected included age, gender of patient and physician, and reason for a visit. Logistic regression was used to test the association between the variables and risk of failing to complete a TBSE.

**Results:**

The risk for omitted TBSE is higher in older patients, females, patients whose primary reason for seeking care was not specifically for a complete skin check, and with female physician. Patients > 80 years had more than four times increased risk of not undergoing TBSE compared to the youngest (< 30 y). The strongest correlation to omitting TBSE was with other reasons for primary care visits than “skin check”. Male gender of the patient and being examined by male physicians decreased the risk of omitted TBSE by 20% and 30%, respectively. There was no evidence of interaction between the gender of the patient and the physician.

**Conclusion:**

Since TDS reduces the opportunities to have a TBSE by dermatologists, the standard management of patients with suspicious skin lesions in primary care must be revised and evidence-based. TBSE is strongly recommended for patients with increased risk of skin cancer, for example old persons with fair skin and a history of skin cancer, when managing them with TDS.

## Introduction

Skin cancer is the most common cancer worldwide, predominantly affecting countries with fair-skinned populations [1]. Compared to the overall cancer incidence, which has decreased globally in recent years, skin cancer incidence increases by 3–10% yearly [2]. In Sweden, the incidence has risen by 5–6% per year over the last decades [3]. Two approaches to identifying skin cancer can be utilized, total body skin examination (TBSE) or lesion-directed screening (LDS). TBSE is a visual exam of the patient’s entire skin compared to LDS, where only a specific lesion is investigated [4]. Previous studies have shown that patients with a high risk of skin cancer benefit most from TBSE [5]. However, TBSE has some disadvantages and possible impediments, such as the requirement of more time and resources and that patients may be uncomfortable in letting a provider examine perceived private body parts [4, 6]. Furthermore, in the literature, there is no consensus guideline on which patients should be examined with TBSE or how it should be performed [7].

Teledermoscopy (TDS), the distant assessment of dermoscopically imaged skin lesions, enhance the diagnostic accuracy and, thereby the efficiency and medical triage of skin cancer care by transferring the dermatologist´s expertise to the primary care setting [8, 9]. There is, however, a risk for undiagnosed skin cancer when managing patients with TDS if TBSE is not performed in connection to the consultation in primary care [10]. Since TDS aims at decreasing referrals for a face-to-face consultation with dermatologists, most TDS-managed patients will not receive a TBSE from a dermatologist. It has previously been shown that more than 25% of incidental skin cancers could be missed if only LDS is used in the TDS management of patients [11]. In a high-risk population, Omara et al. [12] showed that 21.7% of detected skin malignancies in a skin cancer clinic were detected incidentally upon performing a TBSE. Factors that increase the risk of omitting TBSE are largely unknown. Considering that TBSE is more time-consuming than LDS, time constraints might result in down-prioritizing performing TBSE for all patients. Understanding the underlying mechanisms could influence the healthcare system and help primary care physicians to offer evidence-based, high-quality care. For example, primary care is constantly under increasing pressure from caring for older and more complex patients, which might influence the workflow. With increased knowledge, primary care physicians might reassess how they manage patients in connection to TDS, especially in patients with a high risk of skin cancer. Hence, this study aims to elucidate patient and physician characteristics associated with failure of TBSE and put these factors into the perspective of patients at high risk for incidental skin cancers.

## Methods

**Fig. 1:**
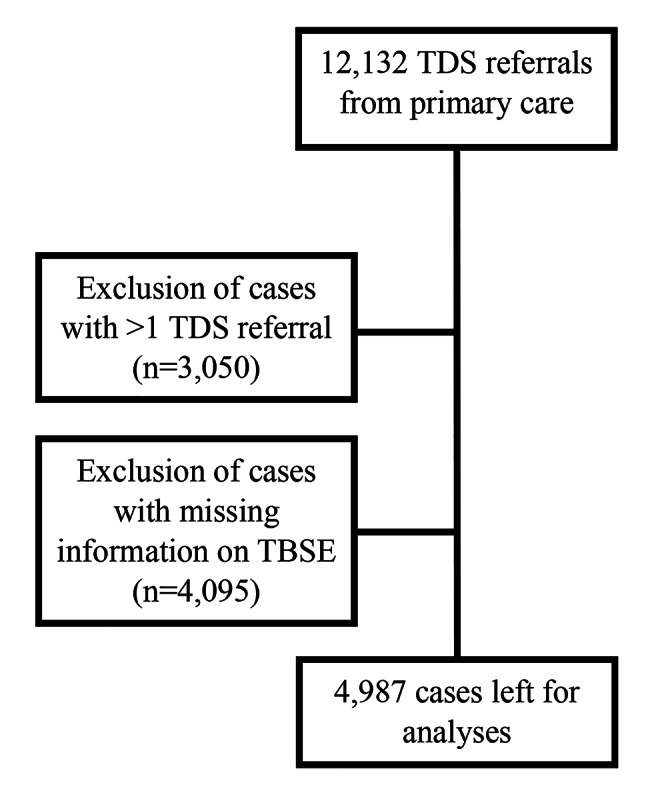
Flowchart depicting selection of final dataset for analyses

This retrospective cohort study used consecutive collected referrals from a TDS referral system (Dermicus®) 2009–2021. Included patients were from Sweden’s capital healthcare region (Region Stockholm). The study was approved by the national Swedish Ethic Review Authority. Only first TDS referral for each person from the primary care centres was used. Cases with missing information on performed TBSE were excluded, see Fig. 1. Data collected included age, gender of patient and physician (extracted from referring physician’s name), localization, reason for visit, TDS diagnosis, histopathological diagnosis, planned action, and year of referral. The TDS referral system had a checkbox to indicate in each referral whether TBSE was performed or not. All primary care centres starting to use TDS had an onboarding session of a couple of hours with a lecture about skin cancer incidence, detection and dermoscopy. In this lecture, the importance of TBSE was emphasized. However, no routine reminders to perform TBSE was given if the primary care physician had not completed it.

Stata version 17 (StataCorp. 2021. *Stata Statistical Software: Release 17*. College Station, TX: StataCorp LLC) was used for statistical analyses. Descriptive statistics were compiled, including comparative statistics between cases with and without information on performed TBSE. Univariate and multivariate logistic regression was used to test the association between the exposure variables and risk for omitting a TBSE. An interaction term was introduced for gender of patient and gender of physician and was included in the final multivariate regression model. A p-value < 0.05 was considered significant.

## Results

12,132 TDS referrals sent from primary care centers were identified. After excluding persons who had > 1 TDS referral (n = 3,050) and cases with missing information on whether TBSE had been performed (n = 4,095) 4,987 TDS referrals were included in the study cohort. Demographics and data on TBSE performance are shown in Table 1. TBSE were performed in more than 70% of all patients but more frequent in males (79.0%) than in females (73.4%). It was more common for younger than older patients to receive TBSE. In persons younger than 30 years 79.4% had a TBSE compared to 46.9% in patients > 80 years of age. The vast majority (94.6%) who visited their physician for a skin/ nevus check had a TBSE. However, if the primary purpose of the visit was either a specific skin lesion or another unrelated matter, TBSE was only performed in just over 60%. Male physicians performed TBSE more frequently (78.5%) than female physicians (73.1%). In Table 1 the difference in patient characteristics depending on available information on TBSE is also shown. Whether or not TBSE was performed was less often recorded for old patients, when the primary reason for the visit was “other visit” and when the physician was male.

**Table 1:** Basic characteristics of persons referred by teledermoscopy according to whether total body skin examinations were performed

Characteristic	TBSE performed, n (%)	TBSE not performed, n (%)	P (chi^2)^	Information on TBSE available, n (%)	Information TBSE missing, n (%)	P (chi2)
**Gender patient**						
Female	2,233 (73.4)	809 (26.6)		3,042 (54,5)	2,541 (45.5)	
Male	1536 (79.0)	409 (21.0)	**< 0.005**	1,945 (55.6)	1,554 (44.4)	0.3
**Age**						
< 30	559 (79.4)	145 (20.6)		704 (56.2)	548 (43.8)	
30–59	2,120 (79.9)	532 (20.0)		2,652 (57.6)	1,956 (42.5)	
60–79	1,000 (69.5)	439 (30.5)		1,439 (51.8)	1,340 (48.2)	
>=80	90 (46.9)	102 (53.1)	**< 0.005**	192 (43.3)	251 (56.7)	**< 0.005**
**Reason for visit**						
Skin check (nevus check)	1,954 (94.6)	112 (5.4)		2,066 (77.3)	606 (22.7)	
Other visit	254 (65.6)	133 (34.4)		387 (38.0)	631 (62.0)	
Specific lesion visit	1,561(61.6)	973 (38.4)	**< 0.005**	2,534 (47.0)	2,858 (53.0)	**< 0.005**
**Gender physician**						
Female	2,022 (73.1)	740 (26.8)		2,762 (58.9)	1,931 (41.2)	
Male	1,747 (78.5)	478 (21.5)	**< 0.005**	2,225 (50.7)	2,164 (49.3)	**< 0.005**

Multivariate analyses (Table 2) revealed that male patients had a slightly smaller risk of omitted TBSE compared to female patients (OR 0.8; 95% CI 0.7–0.9). With increasing age, the risk of a person not receiving a TBSE rose continuously. Individuals > 80 years had more than four times higher risk than younger individuals (< 30). Physicians of male gender were slightly more likely to perform TBSE compared to female physicians (OR 0.7; 95% CI 0.6–0.8). There was no statistically significant interaction between the gender of the patient and the gender of the physician. Interestingly, the lowest risk of not having a TBSE, was with a male patient-male physician combination (OR 0.5; 95% CI 0.4–0.6). Most impact on the likelihood of examining the entire skin was the primary reason for the visit in primary care. Compared to seeking medical care for nevus/ skin check, people who visited for another reason had 8.2 (95% CI 6.0-11.1) times increased risk, and people visiting to get an assessment for one specific skin lesion had a 10.4 (95% 8.4–12.9) times increased risk of omitted TBSE.

**Table 2:** Odds ratio (OR) and 95% confidence intervals (CI) for risk of not performing total body skin examinations (TBSE) in primary care in connection to teledermoscopy referrals estimated using univariate and multivariate logistic regression

Risk factor	Univariate logistic regression OR (95% CI)	p-value	Multivariate logistic regression OR (95% CI)	p-value
**Gender patient**				
Female	Reference		Reference	
Male	0.7 (0.6–0.8)	**< 0.005**	0.8 (0.7–0.9)	**0.001**
**Age patient**				
< 30	Reference		Reference	
30–59	1.0 (0.8–1.2)		1.1 (0.9–1.3)	0.5
60–79	1.7 (1.4–2.1)		1.8 (1.4–2.2)	**< 0.005**
>=80	4.5 (3.2–6.3)	**< 0.005**	4.4 (3.0-6.4)	**< 0.005**
**Gender physician**				
Female	Reference		Reference	
Male	0.7 (0.6–0.8)	**< 0.005**	0.7 (0.6–0.8)	**< 0.005**
**Gender Combinations physician and patient**				
Female physician-female patient	Reference		Reference	p for interaction 0.55
Female physician- male patient	0.8 (0.6–0.9)		0.8 (0.7-1.0)	**0.03**
Male physician – female patient	0.8 (0.6–0.9)		0.7 (0.6–0.9)	**< 0.005**
Male physician – male patient	0.6 (0.5–0.7)	**< 0.005**	0.5 (0.4–0.6)	**< 0.005**
**Reason for visit**				
Skin check/ nevus check	Reference		Reference	
Specific for one skin lesion	10.6 (8.6–13.0)		10.4 (8.4–12.9)	**< 0.005**
Visit for other reason	9.6 (7.2–12.9)	**< 0.005**	8.2 (6.0-11.1)	**< 0.005**

OR = Odds ratio

CI = Confidence interval

## Discussion

We conducted a retrospective cohort study to better understand the underlying factors for not performing TBSE in connection to TDS consultations in primary care. Not surprisingly, the reason that brought the patient to seek medical care impacted the risk of omitted TBSE most. Unfortunately, the risk of omitted TBSE was increased not only when the visitor’s primary purpose was non-skin related but also when patients sought consultation concerning one specific skin lesion. Furthermore, high age of the patient and female gender had a negative effect on the probability of receiving a TBSE.

The importance of performing TBSE is emphasized in several previous studies. Aldrige et al. [13] found through analysis of two prospective cohorts of patients referred from primary care to dermatology clinics, that more than 30% of detected melanomas were found upon TBSE. Similarly, a prospective, population-based cohort study in Australia found that 12% of all melanomas were incidentally detected and 35% were detected through routine skin checks [14]. In two studies from dermatology hospital clinics in Spain and the UK, as much as 26.6% and 21.7% of all detected skin cancers were detected incidentally [12]. However, these results might not be entirely applicable to the primary care setting, in which the populations are not selected on skin cancer risk. In a study from Belgium, the skin cancer detection rate per 100 screened participants did not differ between TBSE (2.3%), and LDS (3.2%) and LDS was found to be 5.6 times less time consuming [4]. Studies have also been performed on patients referred by TDS and found that 3.6–15.6% were found to have incidental skin cancers upon a face-to-face follow-up. The risk of missing skin cancers might also vary with different methods used for dermatologic teleconsultations. For example, real-time video teledermatology sessions compared with store-and-forward TDS referrals give different information. However, both these methods have in common that they will result in fewer TBSEs performed by dermatologists and thereby likely lead to a higher risk of missed skin cancer. Risk factors for having incidental skin cancers include older age (> 50), male gender, light skin phototype, having many nevi, living alone, lesion on the trunk, family history of skin cancer, personal history of skin cancer [11, 12, 15, 16].

With increasing age, this study found that the risk of omitting TBSE increased. This effect appeared in people over 59 years old, and in people over 80 years this risk was increased by 4.5 times compared to persons younger than 30 years. Furthermore, from our analyses of the referrals that lacked information on performed TBSE, this was more common for older patients, suggesting this might be an even greater risk factor than indicated by our results. In several previous skin cancer risk models, older age has been shown to be a significant factor [17, 18]. Therefore, it is particularly unfortunate that this patient group is at a higher risk of not receiving TBSE in primary care, especially in the setting of TDS which results in fewer TBSEs performed by dermatologists. We had no information on why a TBSE was more often omitted for older people. Speculatively, it might be due to time limitations since, on average, it takes longer time for older people to get undressed. To avoid missing skin cancers, this unfavorable situation must be addressed to stress the importance of TBSE already at the primary care level when managing older persons with TDS.

A performed TBSE includes examination of private body parts and the different gender combinations can influence if a TBSE is performed and whether examination of sensitive body parts is included in the TBSE. Kuraitis et al. found in a survey study of dermatology residents in America that male residents had lower exposure during training to female genital examinations, which might impact their future practice [19]. Their finding is in contrast with our study, which found that male physicians more often performed TBSE. There was neither a great impact on physician-patient gender discordance, even though the chance of having a TBSE was highest for male persons that was examined by male physicians. Importantly, male physicians were more likely not to record whether they had performed a TBSE. Therefore, these results should be cautiously interpreted. The underlying explanation for these gender differences is also difficult to discern and needs further study.

Not surprisingly, the primary reason for the visit to primary care significantly impacted the probability of getting a TBSE. We had no specific information about the possible influence of time pressure on primary care physicians during the visit that generated the TDS referral. However, the time pressure was probably higher in visits booked for other reasons than a specific skin lesion/ skin check. Time constraints are a substantial problem for most primary care physicians, with several underlying mechanisms. Firstly, the prevalence of multimorbidity and complexity of patients visiting primary care is high. In a prior study, nine out of ten persons had more than one chronic condition and the prevalence of multimorbidity increased with age [20]. Secondly, the organization of primary care with different financial incentives to reduce hospital-based health care, impacts primary care in different ways. In Sweden, the primary health care system was reformed in 2009, introducing a free choice of provider for the population in combination with freedom of establishment for private primary care providers [21]. Gradually all regions in Sweden now have a reimbursement system promoting that all health issues of a patient are managed during as few visits as possible. As a consequence, Swedish primary care physicians tend to deal with skin lesions brought to their attention during a visit for other primary purposes. Presumably, this results in too little time to perform TBSEs. In addition, to save time and if patients seek advice in primary care for one specific skin lesion, traditionally the standard care has been only to examine this particular lesion. In an era of increasing management of patients through e-health, the usual methods of examining patients must be re-evaluated and evidence-based. Failure to perform TBSE in primary care, especially in the setting of TDS, carries an obvious risk of missing skin cancer. This risk is particularly unfavourable for patients at high risk of skin cancer, for example persons with fair skin types, older age, male gender, living alone, prior skin cancer (including a malignant index skin lesion), severely sun damaged skin, many nevi, and heredity for skin cancer. However, suggesting performing a TBSE for all patients managed with TDS would not be cost-effective. We therefore argue that consideration of risk factors for incidental skin cancer should be taken into account when managing patients with TDS, to determine the need for TBSE. For high-risk patients (for example older patients with a suspicious index lesion or a history of skin cancer) that ask for an assessment of a skin lesion during a visit for another primary reason (such as hypertension), primary care physicians might serve the patient better by setting up a new visit that allows for TBSE in conjunction with TDS. These secondary effects on health care systems of implementing tele-health services, such as performing TBSE in primary care instead of in specialized dermatology care, should be considered during the implementation’s planning phase. Required resource allocation to the affected stakeholders should be analysed before implementing the system.

### Strengths and limitations

The main strength of our study is the extensive data set covering twelve consecutive years, including both genders and all ages. Our main limitations include the large proportion of missing information on performed TBSE and the lack of information on other risk factors for skin cancer. Also, there was no information about the reason for not performing TBSE. Hence, we do not know if the patient declined a TBSE or if time was the limiting factor. Furthermore, there is no internationally accepted practice on how to perform a TBSE. Currently, each physician develops their personal way of achieving a TBSE. Therefore, we could expect variations in how comprehensive a registered TBSE is, not only in this study. [7] We might also have had some selection bias in which persons were more likely to bring the primary care physician’s attention to a suspicious skin lesion. Alike prior studies investigating utilization of skin checks for skin cancer prevention, it is probable that there will be a higher proportion of young women, with a higher than average education level, as well as fair-skinned people attending these clinics [22]. However, we do not think this possible skewed selection of participants will have impacted the association between investigated risk factors and probability of performing a TBSE.

## Conclusion

This study found a high risk of omitted TBSE in older persons, females, and persons whose primary reason for seeking care was not a skin check. Failure to perform TBSE when managing patients with TDS is particularly unfavourable for patients at high risk of skin cancer. Hence, it is crucial for primary care physicians to consider risk factors for incidental skin cancer when managing patients with TDS. The shift in responsibility to perform TBSE from secondary to primary care should be recognized in resource allocation when implementing TDS. The results of this study can be used to develop and improve the use of TDS. For example, a pop-up reminder to perform TBSE could be implemented in the TDS application when a high-risk person is identified by added information on risk factors.

## Data Availability

The dataset used during the current study is available from the corresponding author on reasonable request.

## Abbreviations

TBSE: Total body skin examination
TDS: Teledermoscopy
LDS: Lesion-directed screening
OR: Odds ratio
CI: Confidence intervals
